# MicroRNA-663 inhibits the proliferation, migration and invasion of glioblastoma cells via targeting TGF-β1

**DOI:** 10.3892/or.2022.8324

**Published:** 2022-05-03

**Authors:** Qizhuang Li, Quan Cheng, Zigui Chen, Renjun Peng, Rui Chen, Zhiming Ma, Xin Wan, Jincan Liu, Ming Meng, Zhigang Peng, Bing Jiang

Oncol Rep 35: 1125–1134, 2016; DOI: 10.3892/or.2015.4432

Subsequently to the publication of the above article, an interested reader drew to the Editors attention that they had identified several instances of overlapping data panels comparing between the scratch-wound assay data (‘36 h’ experiments) portrayed in Figs. 3 and 8; furthermore, there appeared to be an overlap in a pair of the data panels shown for the Transwell assay experiments with U87 cells in Fig. 9 (albeit with an inversion of one of the panels), such that these data may have been derived from the same original source, even though they were purportedly intended to show the results from differently performed experiments.

Given the multiple instances of overlapping data panels that have been identified in the compilation of the figures in this article, the Editor of *Oncology Reports* has decided that this article should be retracted from the publication on account of a lack of overall confidence in the presented data. The authors were asked for an explanation to account for these concerns, but the Editorial Office did not receive any reply. The Editor apologizes to the readership for any inconvenience caused.

